# Large, calcified aneurysm of the left ventricle: Case report of an incidental finding

**DOI:** 10.1002/ccr3.6160

**Published:** 2022-07-25

**Authors:** Aleksandar Georgiev, Silvia Tsvetkova, Georgi Goranov, Petar Nikolov

**Affiliations:** ^1^ Department of Diagnostic Imaging Medical University Plovdiv Plovdiv Bulgaria; ^2^ Department of Cardiology Medical University Plovdiv Plovdiv Bulgaria

**Keywords:** acquired, CT, diagnostic imaging, hearth, myocardial infarction, vascular

## Abstract

The case is of an 88‐year‐old female patient with an accidental finding of a large, calcified aneurysm near the cardiac apex. Differential diagnoses can be made with false aneurysms and congenital diverticulums. Imaging modalities beneficial for diagnosing LVA are ultrasound, X‐rays, CT, MRI, including PET/CT for oncology patients.

## Graphical abstract

Left ventricular aneurysms are most often a consequence of myocardial infarction, which is sometimes occult, then they are presented as accidental imaging finding visible with different modalities.

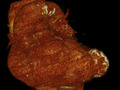

## 1 CASE PRESENTATION

**FIGURE 1:**
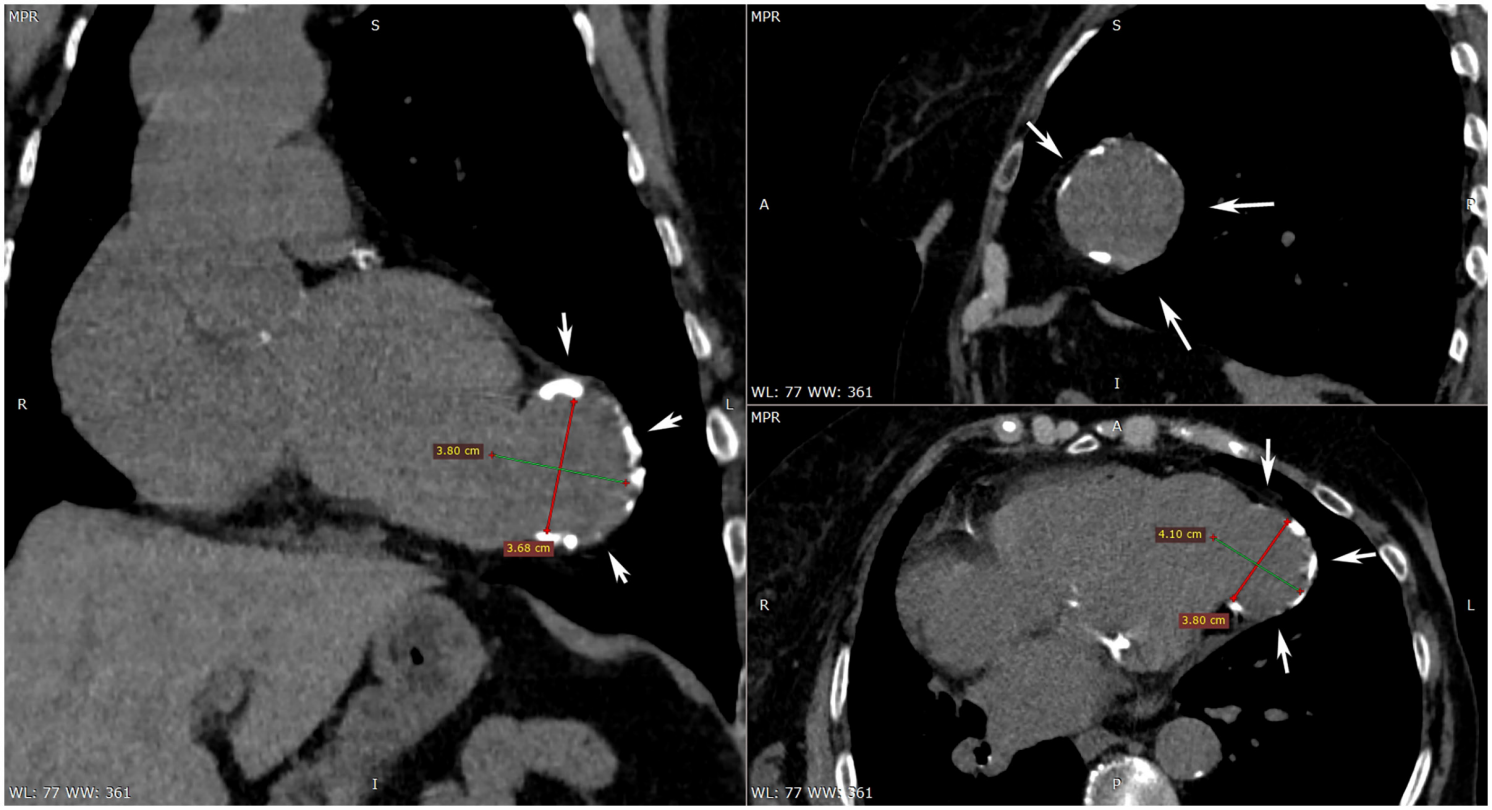
CT images of the aneurysm in oblique, sagittal, and axial view, with diameter measurements

The presented case is of an 88‐year‐old female patient with breast cancer. The staging CT scan reveals an accidental finding of a large, calcified aneurysm near the cardiac apex. The formation is rounded in shape and approximately 4 cm in diameter (Figure 1, Video S1). CT shows severe calcification of the left anterior descending (LAD). Therefore, the patient has evident occult coronary artery disease. The anterior myocardial infarction probably occurred “silent”—a long time ago. After the finding, the patient refused consultation with a cardiac surgeon and opted for conservative therapy. Aneurysms in the left ventricle (LVA) usually appear after myocardial infarction.^1^, ^2^ Imaging modalities beneficial for diagnosing LVA are ultrasound, X‐rays, CT, and MRI. Hybrid methods such as PET/CT could be used for the diagnosis in the context of oncology staging and restaging. Clinical symptoms may include angina or dyspnea due to systolic and diastolic dysfunction, ventricular arrhythmias leading to syncope, palpitation, heart failure, or sudden death.^1^, ^2^ Thromboembolic events (stroke, acute limb ischemia, or MI) are uncommon.^1^, ^2^ Differential diagnoses can be made with false aneurysms and congenital diverticulums. About 80% of LVA are located in the anterior or apical wall, most commonly associated with LAD artery occlusion,^1^, ^2^ such as in the presented case.

## AUTHOR CONTRIBUTIONS

Aleksandar Georgiev is responsible for image evaluation and drafting of the manuscript. Silvia Tsvetkova is responsible for image and critical assessment. Georgi Goranov is responsible for data interpretation and patient consultation. Petar Nikolov is responsible for data interpretation and critical assessment.

## CONFLICT OF INTEREST

The authors declare no conflict of interest.

## CONSENT

Written informed consent was obtained from the patient to publish this report in accordance with the journal's patient consent policy.

## Supporting information


Video S1

## Data Availability

The data that support the findings of this study are available on request from the corresponding author. The data are not publicly available due to privacy or ethical restrictions.
